# Irisin in Pediatric Obesity: A Narrative Review of Current Evidence

**DOI:** 10.7759/cureus.111456

**Published:** 2026-06-24

**Authors:** Zuzanna Pajak, Natalia Wizner, Katarzyna Kania, Zofia Korzonkiewicz, Teresa Wojtowicz, Nikoletta Rauer, Krzysztof Zapart, Milosz Janikowski

**Affiliations:** 1 Faculty of Medicine, Medical University of Silesia, Katowice, POL; 2 Department of Psychiatry and Psychotherapy of Developmental Age, Medical University of Silesia, Katowice, POL; 3 Faculty of Medicine, Medical University of Gdańsk, Gdańsk, POL

**Keywords:** adolescence, adolescent, child, children, fndc5, irisin, obesity, pediatric

## Abstract

Obesity in children and adolescents represents an escalating global health challenge associated with chronic low-grade inflammation and dysregulation of myokine and adipokine secretion. Irisin, encoded by the FNDC5 gene, plays a pivotal role in energy homeostasis by promoting the “browning” of white adipose tissue (WAT) and increasing energy expenditure through thermogenesis. The aim of this study was to establish the diagnostic and therapeutic significance of irisin in the context of childhood obesity and to assess its usefulness as a marker for the early detection and monitoring of metabolic disorders. A comprehensive literature search was conducted using the PubMed database. Findings were limited to studies published within the last five years, from April 14, 2021, to April 14, 2026. Only studies in English were included. We used the following keywords and phrases: irisin; FNDC5; obesity; child; children; pediatric; adolescence; adolescent. Twenty studies met the inclusion criteria and were included in this narrative review.

Studies confirmed that serum irisin concentration typically correlates positively with BMI and leptin levels, which may be described as a compensatory mechanism for improving insulin sensitivity. In obese adolescent girls with polycystic ovary syndrome (PCOS), irisin levels are lower but increase with a reduction in fat mass. High-intensity interval training (HIIT) stimulates higher irisin release than moderate-intensity training. In rarer conditions, such as Prader-Willi syndrome (PWS), irisin levels are reduced, which is associated with impaired bone metabolism and decreased muscle mass. Irisin can also act as a marker of hepatic steatosis and as a factor supporting executive functions in overweight children. Irisin represents a promising and sensitive biomarker of metabolic status and adipose tissue content in pediatric patients. Its monitoring may allow for a clear assessment of therapeutic effectiveness and dynamic changes in the child’s metabolic profile.

## Introduction and background

Obesity in children and adolescents represents one of the greatest global challenges in modern medicine. It is a major risk factor for the development of numerous chronic diseases, such as hyperlipidemia, type 2 diabetes, arterial hypertension, and metabolic syndrome, which often persist into adulthood [1]. The prevalence of obesity in children and adolescents has increased more than fourfold over the last four decades, and the WHO indicates that this problem affects approximately 340 million children and adolescents aged five to 19 years worldwide [2-3]. The etiology of obesity is complex, yet a key element in its development remains the prolonged dysregulation of the body’s energy balance, involving cytokines derived from adipose tissue (AT; adipokines) and skeletal muscle (myokines). Recent interest in these molecules has contributed to an increased volume of scientific research on their role [4-5]. A crucial aspect of obesity pathophysiology is chronic low-grade inflammation, which is sustained by AT itself. The development of obesity can be characterized by macrophage infiltration into AT, leading to the release of pro-inflammatory cytokines, including IL-6 and TNF-alpha, and activation of pro-inflammatory pathways such as JNK and NF-kappaB [5].

Irisin was discovered in 2012 and described as a hormone-like myokine-adipokine. The precursor of this molecule is encoded by the FNDC5 gene, which is expressed in key organs such as the liver, heart, and brain [6]. Irisin is released mainly by skeletal muscle during exercise and fasting, as well as by AT. This molecule has been proposed as a regulator of energy homeostasis, although the magnitude and clinical relevance of this role in humans remain under investigation. It is an adipomyokine that is a fragment of the extracellular domain of the FNDC5 protein and is released in response to physical activity and stimulation of peroxisome proliferator-activated receptor-gamma coactivator 1-alpha (PGC-1-alpha) [7]. Experimental and clinical studies have suggested that irisin may be involved in thermogenesis, UCP-1-related browning of white adipose tissue (WAT), and metabolic adaptation to exercise [8-11]. However, most pediatric data remain observational, and irisin should not be regarded as an established therapeutic target or validated clinical biomarker at present. Although the existence and physiological role of irisin have been debated, particularly regarding antibody specificity, circulating irisin quantification, and proteolytic cleavage of FNDC5, accumulating evidence supports its relevance as a potential metabolic mediator. Readers should nevertheless be aware of these methodological concerns when interpreting studies on circulating irisin [12-15].

## Review

Search strategy

A structured literature search was conducted using the electronic PubMed database to identify studies examining circulating irisin or FNDC5 in pediatric obesity and related metabolic disorders. The search was conducted on April 14, 2026, and was limited to studies published in English within the preceding five years, from April 14, 2021, to April 14, 2026, in order to ensure the current relevance of the findings. The following PubMed search formula was used: ((irisin[Title/Abstract]) OR (FNDC5[Title/Abstract])) AND ((obesity[Title/Abstract]) OR (overweight[Title/Abstract]) OR ("metabolic syndrome"[Title/Abstract]) OR ("insulin resistance"[Title/Abstract])) AND ((child*[Title/Abstract]) OR (adolescent*[Title/Abstract]) OR (pediatric[Title/Abstract]) OR (paediatric[Title/Abstract])). Reference lists of relevant articles were additionally screened to identify further eligible sources. No searches were performed in Embase, Scopus, Web of Science, gray literature databases, or trial registries; this restriction is acknowledged as a limitation.

Study selection

**Figure 1:**
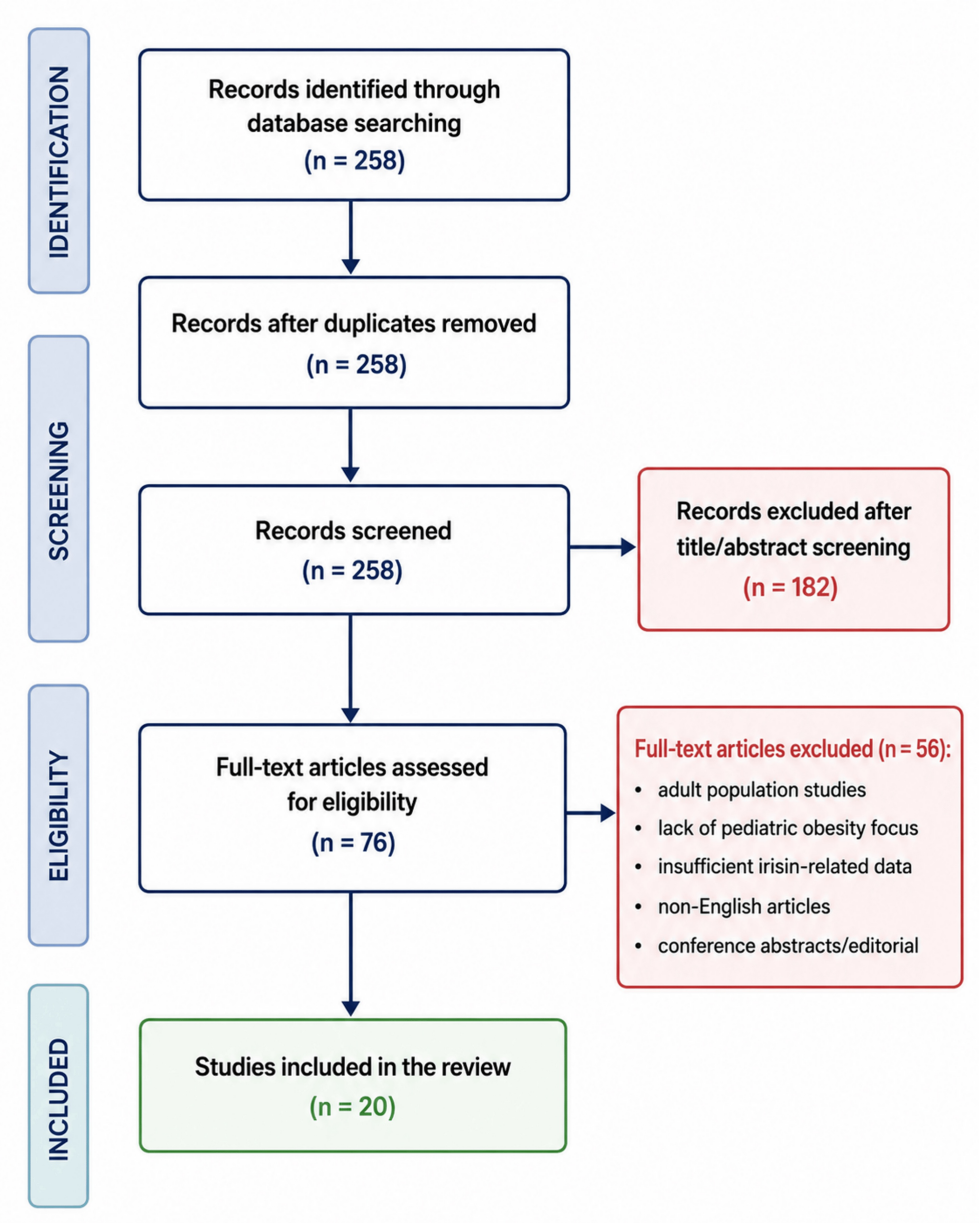
PRISMA-inspired flowchart illustrating the process of study identification, screening, eligibility assessment, and inclusion in the present narrative review. PRISMA: Preferred Reporting Items for Systematic Reviews and Meta-Analyses.

The selection process involved a thorough assessment of titles, abstracts, and full texts, with two reviewers independently evaluating each study. Any disagreements regarding eligibility were resolved based on the predefined inclusion criteria through discussion and consensus. No automated tools were utilized at any stage of the selection process. Inclusion criteria comprised original research studies, including cross-sectional, case-control, cohort, and interventional designs, conducted in pediatric or adolescent populations (≤18 years of age), assessing serum or plasma irisin concentrations in relation to obesity, body composition, metabolic parameters, or related clinical conditions. Exclusion criteria included articles not written in English, letters to the editor, conference abstracts, studies on animal models, studies focusing exclusively on adult or mixed-age group populations, and articles without full-text availability. The search identified 258 records. After title and abstract screening, 76 full-text articles were assessed for eligibility, and 20 studies that met the inclusion criteria were ultimately included in the present narrative review. Because the search was conducted in a single database, no duplicate records from overlapping databases were expected; nevertheless, records were manually checked for duplicates before screening. Study identification and selection are summarized in a revised PRISMA-inspired flowchart. The flowchart now reports the number of records identified, screened, excluded, assessed for eligibility, and included, together with the main reasons for exclusion at the eligibility stage (Figure 1).

**Figure 2:**
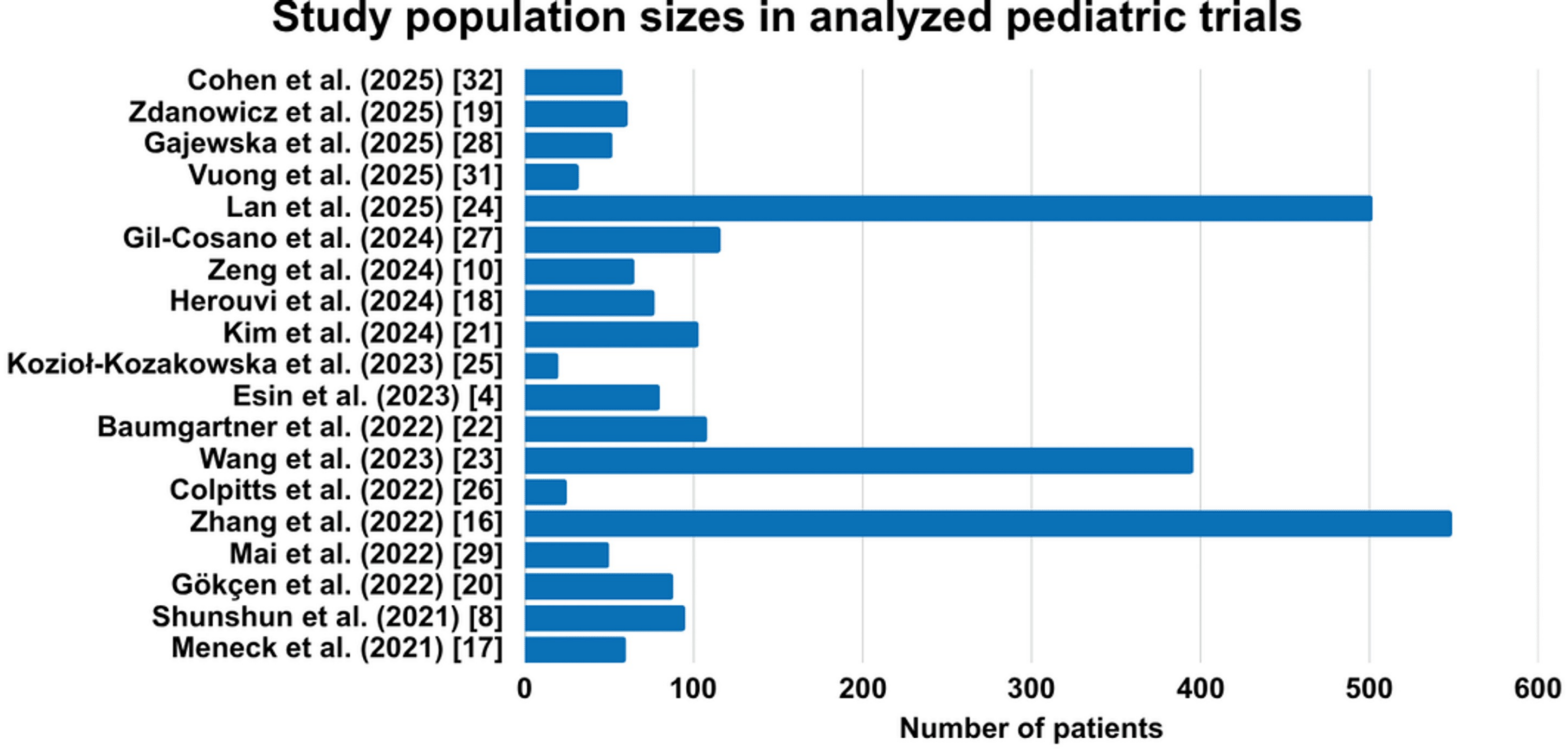
Comparison of sample sizes, expressed as the number of pediatric participants, across the largest analyzed studies included in the narrative review.

Due to the narrative nature of this review, no formal risk-of-bias tool or systematic quality appraisal was applied. However, key methodological characteristics of the included studies, including study design, sample size, single-center or multicenter setting, control group, adjustment for confounders, and irisin quantification method, were considered descriptively when interpreting the evidence. The study population sizes of the included pediatric studies are summarized in Figure 2.

Thematic synthesis

The included studies were synthesized using a thematic approach, in which the principal findings were grouped into clinically meaningful domains reflecting the multidirectional role of irisin in pediatric obesity. The following themes were identified and form the structure of the subsequent sections: (1) irisin as a molecule associated with energy homeostasis in childhood obesity, including its relationship with BMI, leptin, and adipose tissue; (2) irisin in endocrine and metabolic dysfunction, including central precocious puberty (CPP) and PCOS; (3) the role of irisin in musculoskeletal health and hepatic disorders, with particular emphasis on metabolic dysfunction-associated steatotic liver disease (MASLD); (4) genetic polymorphisms of the FNDC5 gene and the influence of irisin on cognitive performance; (5) the impact of physical activity, including acute and chronic exercise, on the irisin profile; and (6) irisin dynamics in rare genetic and chronic metabolic conditions, such as Prader-Willi syndrome (PWS) and type 1 diabetes mellitus (T1DM). For each theme, key quantitative findings, methodological characteristics of the included studies, and clinically relevant interpretations were extracted and integrated narratively. Because no formal quality appraisal was performed, correlation coefficients, p-values, and proposed thresholds are presented as study-level observations and should be interpreted cautiously.

Beyond BMI: Irisin and energy homeostasis in childhood obesity

The relationship between serum irisin concentration and pediatric obesity is highly complex [16-18]. A 2022 study reported that BMI may not affect irisin directly, but rather through leptin pathways [16]. In a study involving 366 students aged eight to 15 years, BMI significantly influenced leptin concentrations across all analyzed weight categories, and leptin was reported as an independent predictor of irisin. The leptin-irisin axis was particularly pronounced in girls, with standardized path coefficients of β = 0.75 in the normal-weight group and β = 0.73 in the overweight and obese group (p < 0.01). In boys, these correlations were β = 0.60 and β = 0.64, respectively (p < 0.01). These findings suggest a close interaction between both molecules in signaling energy reserves to the hypothalamic-pituitary-gonadal axis. Concurrently, hormonal disruptions in advanced obesity, such as hyperleptinemia and adiponectin deficiency, are strongly associated with insulin resistance [4]. Children with insulin resistance exhibited nearly threefold higher leptin concentrations (16.4 ± 8.25 vs. 5.5 ± 3.28 ng/mL, p = 0.000) and significantly lower adiponectin concentrations (6.2 ± 1.79 vs. 8.0 ± 2.56 μg/mL, p < 0.05) compared to peers with normal insulin sensitivity [4].

However, existing reports regarding absolute irisin concentrations in children remain partially discrepant. Some studies reported a positive correlation between irisin and body adiposity, with associations observed for age (ρ = 0.327; p = 0.011), BMI (ρ = 0.707; p < 0.001), waist circumference (ρ = 0.624; p < 0.001), total cholesterol (ρ = 0.361; p = 0.044), triglycerides (ρ = 0.419; p = 0.001), and LDL cholesterol (ρ = 0.381; p = 0.003) [17-18]. Children with obesity aged four to 12 years were reported to have higher serum irisin concentrations than controls (p = 0.003), although no significant difference was observed between obesity and extreme obesity subgroups (p = 0.327) [18]. Conversely, other data suggest that serum irisin concentrations may remain constant regardless of body weight. In one cohort of children aged six to 10 years, mean irisin concentrations were similar in children with obesity and normal-weight controls (7.2 ± 1.99 ng/mL vs. 7.1 ± 1.87 ng/mL) and did not significantly depend on sex, vitamin D concentration, or HOMA-IR (p > 0.05) [4]. Differences in age, pubertal stage, sex distribution, obesity severity, BMI classification methods, physical activity patterns, metabolic status, and ELISA kits used may explain the conflicting results regarding the association between irisin and BMI. These sources of heterogeneity limit direct comparison between studies. Standardization of irisin assays and harmonization of study protocols are needed to clarify whether circulating irisin is consistently associated with adiposity in pediatric populations.

Irisin and endocrine/metabolic dysfunction: exploratory evidence

Beyond classical metabolic assessment, available pediatric data suggest that circulating irisin may be associated with selected endocrine and metabolic phenotypes, including CPP and PCOS. However, these findings remain exploratory and should not be interpreted as evidence that irisin is a validated diagnostic tool. In boys with CPP, evaluated in a cohort of 32 patients and 33 healthy peers, irisin concentrations were reported to correlate with BMI, reaching peak values in cases of coexisting obesity [10]. A cutoff value greater than 93.09 ng/mL was reported to identify CPP with 100% specificity in normal-weight boys in this single small study; however, this value should be considered exploratory and study-specific. Excess adiposity reduced the reported diagnostic sensitivity to 47.6%, indicating that irisin, if studied further, may represent, at most, a complementary indicator rather than an independent diagnostic marker. Despite the lack of a direct association with testicular volume or testosterone levels, irisin levels correlated significantly with bone age and the BA-CA differential, suggesting a possible association with biological maturation and skeletal development that requires future confirmation.

A similar overriding influence of adiposity on the irisin profile, albeit leading to a different tissue pattern, was observed in adolescent girls aged 12 to 18 years with PCOS [8]. In a comparative study of 52 patients with PCOS and 43 obese peers without the syndrome, serum irisin concentrations were lower in the PCOS group (256 ± 22.40 vs. 375 ± 18.65 ng/mL; p < 0.05), despite higher BMI and body fat percentage. A one-year weight-reduction program in girls with PCOS was associated with decreased fat mass and increased irisin concentrations (from 256 ± 22.40 to 325 ± 31.74 ng/mL; p < 0.05) [8]. These findings suggest that changes in irisin may accompany improvement in metabolic status after weight management, but they do not establish irisin as a validated biomarker of treatment efficacy. A comprehensive summary of specific diagnostic thresholds, baseline tissue paradoxes, and post-intervention irisin dynamics across these endocrine dysfunctions is presented in Table 1.

**Table 1:** Diagnostic thresholds and interventional dynamics of irisin in endocrine disorders. Source: References [8, 10].

Clinical condition	Diagnostic/cutoff value	Baseline tissue paradox	Interventional outcome and dynamics	Clinical utility and limitations
Idiopathic central precocious puberty (CPP) [10]	>93.09 ng/mL	Obesity-induced excess adipose tissue masks the physiological differences associated with puberty.	Yields 100% specificity for identifying CPP only in boys with a normal BMI. Sensitivity drops to 47.6% overall.	Exploratory complementary indicator only. This cutoff requires external validation in larger independent cohorts before clinical application.
Polycystic ovary syndrome (PCOS) with obesity [8]	N/A (comparative baseline)	Paradoxical suppression: irisin is significantly lower in patients with PCOS (256 ± 22.40 ng/mL) compared to obese peers without PCOS (375 ± 18.65 ng/mL; p < 0.05), despite higher BMI and fat mass.	One-year weight management: fat mass decreased from 46.17 ± 2.33 to 40.25 ± 3.14 kg; irisin level increased from 256 ± 22.40 to 325 ± 31.74 ng/mL (p < 0.05).	Suggestive association with metabolic improvement, but not a validated therapeutic biomarker.

Irisin in musculoskeletal health and liver disease

Beyond endocrine disorders, irisin has been investigated in pediatric hepatology and musculoskeletal health, but current evidence remains preliminary and largely associative. In children and adolescents with MASLD, circulating irisin concentrations have been associated with hepatic steatosis severity [19]. Lower circulating concentrations of this myokine have been linked to more advanced hepatic involvement, supporting its potential role as a non-invasive marker that complements imaging-based assessment of liver involvement in metabolically burdened children. These observations require validation in larger cohorts and with standardized assays before clinical interpretation of absolute concentrations is possible.

Consistent observations have been reported for the broader spectrum of non-alcoholic fatty liver disease (NAFLD), where serum irisin concentrations in pediatric patients reflect both obesity status and hepatic involvement [20]. In obese children and adolescents, circulating irisin has been associated with anthropometric, metabolic, and bone-related parameters, with particular relevance to indices of bone turnover and body composition [21]. Reduced myostatin levels and a more favorable irisin profile have additionally been observed in pediatric patients with adequate vitamin D status, whereas severe pediatric obesity is associated with a myokine imbalance unfavorable for muscle and bone homeostasis [22]. Because irisin physiologically stimulates osteoblastogenesis and limits osteoclast differentiation, its altered secretion in obesity may contribute to disturbances in bone turnover and to the variable bone phenotype observed in this population. The role of irisin in bone-related biomarkers, including bone-specific alkaline phosphatase (BALP) and periostin, further supports its position at the interface between muscle and skeleton, although direct clinical implications remain uncertain [22].

Genetic polymorphisms and irisin-associated cognitive findings

The relationship between FNDC5 genetic variation, irisin, and pediatric obesity has been explored in case-control data [23]. The rs16835198 polymorphism of the FNDC5 gene was reported to modulate the risk of childhood obesity. The presence of the T allele, particularly within the TT genotype, was associated with a higher likelihood of overweight, whereas the GG genotype appeared protective (OR = 0.45). The GT genotype predisposed mainly boys to obesity (OR = 1.68), while the impact of the TT genotype was most pronounced in girls (OR = 3.82) [23].

Parallel to genetic predispositions, circulating irisin has been investigated in relation to neurobiological processes and a potential muscle-brain axis in pediatric populations. A study evaluating 502 children aged seven to 12 years analyzed the relationship between serum myokine concentrations and executive function performance [24]. Although no direct correlation was found between irisin levels and cognitive test scores in the general pediatric population, weight status modified this relationship (p < 0.05). Among overweight or obese children, higher irisin concentrations correlated with better Stroop test indicators, including shorter reaction times (beta = -0.129, 95% CI: -0.228 to -0.030) and reduced verbal interference times (beta = -0.088, 95% CI: -0.163 to -0.013) [24]. These data suggest an association between irisin and selected executive-function parameters in overweight or obese children. However, because the evidence is observational, it does not demonstrate that irisin directly supports cognitive development.

How physical activity shapes the irisin profile in pediatric obesity

A collective analysis of available clinical data suggests that the dynamics of irisin release induced by physical activity in pediatric populations depend on the interaction between stimulus type and baseline metabolic status [25-27]. Regular behavioral interventions have been associated with changes in circulating irisin in children with idiopathic obesity. In a crossover study of children with obesity, individualized dietary support and daily activity monitoring were associated with a statistically significant increase in plasma irisin concentrations from 4.8 to 5.1 μg/mL (p = 0.03) [25]. These findings suggest that regularity and behavioral consistency may be relevant to the irisin profile, although causality and clinical significance remain uncertain.

The acute exercise response appears to differ according to exercise intensity and obesity status. In adolescents aged 12 to 18 years, HIIT was associated with a higher percentage increase in irisin from baseline to peak across the entire study population than moderate continuous exercise (p = 0.049) [26]. The increase was observed in both regimens, reaching 27.5% (SD 46.3; p = 0.022) for moderate exercise and 69.5% (SD 81.6; p = 0.001) for HIIT. However, when directly compared with moderate exercise, the HIIT-related increase did not reach statistical significance in some analyses (p = 0.272) [26]. These findings suggest that excess body weight may be associated with an attenuated acute myokine response, but they do not prove an intrinsic defect in muscle secretory capacity. An assessment of physical activity levels in children and adolescents with a history of oncology treatment showed no statistically significant correlations with irisin, sclerostin, or other bone turnover markers [27]. This may indicate that the relationship between exercise and the myokine-bone profile differs in childhood cancer survivors and requires separate evaluation.

Metabolic challenges: Irisin in PWS and type 1 diabetes

Assessing the irisin profile in rare genetic conditions and chronic metabolic diseases provides insights into its possible role across the muscle-skeletal and glucose-insulin axes. PWS, a rare genetic disorder resulting from the lack of expression of paternally inherited genes in the 15q11.2-q13 region, is characterized by a specific body composition in which a marked deficit in muscle tissue is accompanied by excessive adiposity. Due to this reduced muscle mass, pediatric patients with PWS exhibit differences in irisin secretion compared with individuals with idiopathic obesity [28-30]. Comparative studies of the hormonal profile in children with PWS have demonstrated lower baseline serum irisin concentrations relative to healthy peers (p = 0.031), also confirmed in cohorts involving obese subpopulations (21.8 ± 1.6 vs. 25.9 ± 1.1 ng/mL; p < 0.05) [28-29]. Genetic analyses showed that patients with the 15q11-q13 deletion (DEL15) had lower irisin levels than individuals with uniparental disomy (UPD15) (21.8 ± 0.8 vs. 25.8 ± 1.1 ng/mL; p = 0.018), while sex and pubertal stage did not modify this profile [29]. The reduced baseline irisin concentration in patients with PWS is attributed to lower muscle mass and restricted physical activity [30]. Irisin also acts as an adipokine synthesized by subcutaneous adipose tissue, which is supported by positive correlations with body fat percentage, insulin concentration, and Homeostatic Model Assessment of Insulin Resistance (HOMA-IR). PWS cohorts showed a more favorable glycemic profile, including lower insulin concentrations and reduced insulin resistance compared with controls [29-30]. Nonetheless, positive correlations between irisin and glucose metabolism indices were preserved in both PWS and control groups. Reduced irisin synthesis may also be relevant to skeletal outcomes in PWS. Patients with PWS exhibited lower fat-free mass (p = 0.047), higher fat mass (p < 0.001), and decreased osteocalcin levels (p = 0.052) [28]. Irisin concentration correlated positively with BALP (p = 0.025) and negatively with Gla-OC (p = 0.041) and periostin (p = 0.005) [28]. These associations suggest that lower irisin may contribute to altered bone remodeling in PWS, although causal inference is limited by study design and sample size.

In the context of a dynamic response to physical stimuli, a short-term resistance stimulus appears insufficient to modify the irisin profile [31]. A study involving nine children with PWS, 12 with idiopathic obesity, and 11 healthy peers subjected to resistance exercise showed no significant changes in irisin concentration over time or by group allocation (p ≥ 0.580). The lack of changes in irisin may also reflect the small sample size [31]. In T1DM, a multifactorial analysis of 58 participants aged 11 to 20 years found no significant baseline differences in apelin, irisin, IL-6, FGF21, and myostatin between controls and patients with T1DM. After accounting for weekly physical activity, a significant difference in irisin levels was observed between children with short disease duration and those with long-standing diabetes (p = 0.036), with higher concentrations in patients at an early stage after diagnosis [32].

Discussion 

The majority of the analyzed studies demonstrated that circulating irisin levels positively correlate with obesity indices, such as BMI, with higher concentrations noted in patients with obesity [20]. However, these findings should be interpreted as associations rather than proof of a causal relationship. Higher irisin levels observed in some pediatric obesity cohorts may reflect a compensatory response to excess adiposity, altered leptin signaling, or changes in muscle-adipose tissue communication; however, this interpretation remains hypothetical. Although experimental literature links irisin to UCP-1-related browning of white adipose tissue, pediatric observational data show that higher circulating irisin may coexist with adverse lipid profiles and weaker brown adipose tissue activation in response to cold [17]. Another layer of complexity is that the correlation between irisin and BMI may be mediated by other factors, including leptin [16]. It appears that irisin is not merely a simple marker of body weight but part of a broader hormonal network, potentially influenced by FNDC5 genetic variation [23].

An important limitation of the current irisin literature is the lack of standardization in circulating irisin measurement. Commercial ELISA kits differ in antibody specificity, detection limits, calibration procedures, and reported concentration ranges. In addition, discrepancies between ELISA-based measurements and mass spectrometry-based approaches have raised concerns regarding the accuracy and biological validity of reported serum irisin concentrations. These methodological differences limit direct comparisons between studies and reduce the clinical applicability of absolute irisin values or proposed diagnostic thresholds [12-15]. Irisin concentrations reported across studies are not directly comparable due to the lack of standardization of ELISA kits, antibodies, detection limits, reference materials, sample handling procedures, and reported units. This is particularly important because some studies report values in ng/mL, whereas others use μg/mL or different assay-dependent ranges. Therefore, absolute concentration values and cutoff thresholds should not be interpreted as interchangeable across studies [12-15].

In obese adolescent girls with PCOS, baseline irisin concentration was lower than in age-matched obese peers without PCOS and increased after weight reduction [8]. This suggests that irisin may change alongside metabolic improvement, but it does not validate irisin as a clinical biomarker of treatment efficacy. In PWS, lower irisin levels may be linked to reduced muscle mass and altered bone remodeling. Available data suggest a possible link between muscle mass, irisin, and bone-related biomarkers in PWS, but the contribution of irisin to syndromic bone fragility remains uncertain. In the area of behavioral interventions, exercise intensity appears relevant. HIIT was associated with greater percentage irisin release than moderate-intensity exercise in the whole study population [26]. However, responses were attenuated in overweight or obese subgroups, suggesting that obesity status may modify acute irisin dynamics.

Associations between irisin and MASLD severity, pubertal timing markers, and endocrine dysfunction have been reported, but these findings should be considered exploratory. The proposed CPP cutoff value was derived from a single small study and requires external validation in larger independent cohorts before clinical application can be considered [10,19]. In overweight children, higher circulating irisin concentration correlated with better performance in selected executive-function tests, including the Stroop test [24]. This supports the hypothesis of a possible muscle-brain axis but does not demonstrate that irisin acts as a neuroprotective mediator or directly supports cognitive development.

Future research should focus on several priorities: (1) establishing standardized and validated irisin assays with age-, sex-, and puberty-specific reference ranges for pediatric populations; (2) conducting large, multicenter prospective cohort studies to assess whether irisin predicts metabolic complications, insulin resistance, or cardiometabolic risk over time; (3) performing randomized controlled trials to determine whether lifestyle, exercise, or pharmacological interventions that modify irisin levels are associated with improved clinical outcomes; and (4) harmonizing study protocols, obesity definitions, and adjustment for confounders such as pubertal stage, physical activity, and body composition.

Limitations 

This review has several limitations that should be acknowledged. First, the included studies were highly heterogeneous in terms of study design, participant age, pubertal stage, obesity severity, assessed metabolic outcomes, and laboratory methods, which limits direct comparison of findings. Second, the majority of available studies were cross-sectional, frequently single-center, and based on relatively small cohorts, reducing the ability to establish causal relationships and limiting the generalizability of the results.

Another important limitation concerns methodological inconsistencies in circulating irisin measurements. Different studies used various ELISA assays and laboratory protocols, which may contribute to variability in reported irisin concentrations and affect reproducibility across studies. Commercial ELISA kits differ in antibody specificity, detection limits, calibration procedures, sample handling requirements, and reported concentration ranges. Differences between ELISA-based and mass spectrometry-based measurements further limit the accuracy and biological interpretation of circulating irisin values. Furthermore, the physiological role and accurate quantification of irisin remain subjects of ongoing scientific debate. Although accumulating evidence supports irisin as a potential metabolic mediator, readers should interpret absolute concentrations and proposed thresholds with caution because assay standardization and validated pediatric reference ranges are lacking.

The interpretation of exercise-related findings is additionally complicated by differences in training protocols, exercise intensity, duration of intervention, and timing of blood sample collection. The search was restricted to PubMed and English-language publications, which may have introduced selection and language bias and limited the comprehensiveness of the review. Relevant studies indexed exclusively in other databases, such as Embase, Scopus, or Web of Science, as well as gray literature and trial registries, may have been missed. The absence of a systematic quality appraisal means that the strength of evidence for each finding was not formally evaluated. Therefore, reported quantitative results, including correlation coefficients, p-values, and proposed diagnostic thresholds, should be interpreted with caution, and readers should consult the original studies for methodological details. Despite these limitations, the analyzed studies collectively provide valuable insight into the multidirectional role of irisin in pediatric obesity and related metabolic disorders.

## Conclusions

In conclusion, irisin shows promise as an exploratory biomarker in pediatric obesity research, particularly in relation to adiposity, metabolic dysfunction, exercise-related physiology, and selected organ-specific complications. Several studies have reported associations between circulating irisin, BMI, adiposity indices, leptin, and metabolic parameters, but these findings remain heterogeneous and cannot establish causality. In conditions such as PCOS and PWS, altered irisin levels may reflect changes in body composition, metabolic status, and musculoskeletal physiology, although causal mechanisms remain uncertain. Associations with MASLD severity, biological maturation markers, and executive-function parameters have been reported, but current evidence does not support the clinical use of irisin for diagnosis, monitoring, or therapeutic decision-making. Despite its promise as a prospective diagnostic marker, standardization of assays, harmonization of study protocols, establishment of age-, sex-, and puberty-specific reference ranges, and validation in large independent pediatric cohorts are necessary before clinical application can be considered.
